# Protective Effects of Liposomal N-Acetylcysteine against Paraquat-Induced Cytotoxicity and Gene Expression

**DOI:** 10.1155/2011/808967

**Published:** 2011-04-04

**Authors:** Panagiotis Mitsopoulos, Zacharias E. Suntres

**Affiliations:** ^1^Medical Sciences Division, Northern Ontario School of Medicine, Thunder Bay, ON, Canada P7B 5E1; ^2^Department of Biology, Lakehead University, Thunder Bay, ON, Canada P7B 5E1

## Abstract

Paraquat (PQ) is a herbicide that preferentially accumulates in the lung and exerts its cytotoxicity via the generation of reactive oxygen species (ROS). There is no specific treatment for paraquat poisoning. Attempts have been made to increase the antioxidant status in the lung using antioxidants (e.g., superoxide dismutase, vitamin E, N-acetylcysteine) but the outcome from such treatments is limited. Encapsulation of antioxidants in liposomes improves their therapeutic potential against oxidant-induced lung damage because liposomes facilitate intracellular delivery and prolong the retention of entrapped agents inside the cell. In the present study, we compared the effectiveness of conventional N-acetylcysteine (NAC) and liposomal-NAC (L-NAC) against PQ-induced cytotoxicity and examined the mechanism(s) by which these antioxidant formulations conferred cytoprotection. The effects of NAC or L-NAC against PQ-induced cytotoxicity in A549 cells were assessed by measuring cellular PQ uptake, intracellular glutathione content, ROS levels, mitochondrial membrane potential, cellular gene expression, inflammatory cytokine release and cell viability. Pretreatment of cells with L-NAC was significantly more effective than pretreatment with the conventional drug in reducing PQ-induced cytotoxicity, as indicated by the biomarkers used in this study. Our results suggested that the delivery of NAC as a liposomal formulation improves its effectiveness in counteracting PQ-induced cytotoxicity.

## 1. Introduction

Paraquat (PQ) is a herbicide that preferentially accumulates in the lung and exerts its cytotoxic effects via the generation of reactive oxygen species (ROS) [1–3]. Many studies have focused on increasing the antioxidant status in the lung to protect against PQ injury using various antioxidants, including antioxidant enzymes (e.g., SOD), vitamins (e.g., ascorbic acid, *α*-tocopherol), and low-molecular-weight thiol-containing antioxidants (e.g., glutathione (GSH), N-acetylcysteine (NAC)) but the outcomes from such treatments are limited or without success [2–11]. The failure of these antioxidants to seriously modify the toxicity of the herbicide has been attributed mostly to their inability to cross cell membrane barriers and/or to their rapid clearance from cells [3, 4, 6, 7, 10, 11]. In recent years, it has been demonstrated that the encapsulation of antioxidants in liposomes improves their therapeutic potential against oxidant-induced lung damage, including PQ pulmonary toxicity, because liposomes facilitate intracellular delivery and prolong the retention time of entrapped agents inside the cell [3, 6, 12–15].

Liposomes are phospholipid vesicles composed of lipid bilayers enclosing an aqueous compartment. Hydrophilic molecules can be encapsulated in the aqueous spaces, and lipophilic molecules can be incorporated into the lipid bilayers. Liposomes, in addition to their use as artificial membrane systems, are used for the selective delivery of antioxidants and other therapeutic drugs to different tissues in sufficient concentrations to be effective in ameliorating tissue injuries. The relative ease in incorporating hydrophilic and lipophilic therapeutic agents into liposomes, the possibility of directly delivering liposomes to an accessible body site, such as the lung, and the relative nonimmunogenicity and low toxicity of liposomes have rendered the liposomal system highly attractive for drug delivery [3, 12, 16]. 

In the present study, we compared the effectiveness of conventional NAC and liposomal-NAC (L-NAC) against PQ-induced cytotoxicity and examined the mechanism(s) by which these antioxidant formulations conferred their cytoprotection. N-Acetylcysteine is a low-molecular-weight thiol-containing antioxidant with free radical-scavenging properties [3, 17, 18] attributed to the nucleophilicity and redox interactions of its thiol group [17, 18]. Additionally, NAC is a source of cysteine, often the limiting precursor of *de novo* GSH synthesis [17, 19, 20]. Glutathione is an important antioxidant as it is the most abundant nonprotein thiol present in living cells, and its levels are commonly used as an indicator of intracellular antioxidant status. Furthermore, NAC has been shown to influence redox-sensitive cell-signalling and transcription pathways, such as NF-*κ*B (which regulates proinflammatory genes), the p38, ERK1/2, SAPK/JNK, c-Jun, and c-Fos pathways, among others, in a wide variety of different systems [17, 21, 22]. N-Acetylcysteine has been shown to promote cell growth and survival by activating the MAPK pathway in response to ROS-induced injuries (which normally lead to growth arrest and apoptosis) and is able to limit inflammatory processes, such as the release of proinflammatory cytokines [22]. These actions may play a role in its cytoprotective effect. Accordingly, the cytoprotective effects of NAC or L-NAC against PQ-induced cytotoxicity in A549 human lung adenocarcinoma cells were assessed by measuring cellular PQ uptake, intracellular GSH content, ROS levels, mitochondrial membrane potential, cellular gene expression, inflammatory cytokine release, and cell viability. A549 cells possess many important biological properties of the alveolar epithelial type II cell [23, 24] and have been shown to be useful for studying the metabolic and macromolecule processing contributions of alveolar type II cells to mechanisms of drug delivery at the pulmonary epithelium [25].

## 2. Materials and Methods

### 2.1. Cell Culture and Chemicals

Human alveolar type II-like epithelial A549 cells (ATCC no. CCL-185, American Type Culture Collection, Manassas, Va, USA) were maintained in Costar 0.2 *μ*m vent cap cell culture flasks (Corning, Corning, NY, USA) with standard Dulbecco's modified Eagle's medium nutrient mixture F-12 Ham (Sigma-Aldrich, St. Louis, Mo, USA) supplemented with 10% iron-fortified bovine calf serum (SAFC Biosciences, Lenexa, Kan, USA), 2 mM l-glutamine (Gibco, Carlsbad, Calif, USA), and antibiotic/antimycotic (100 U/mL penicillin, 100 *μ*g/mL streptomycin, and 0.25 *μ*g/mL amphotericin B; Gibco). Cultures were incubated at 37°C in a humidified atmosphere of 5% CO_2_ in air and subcultured when 80% confluent. Prior to plating, cell counts and viabilities were assessed using a Vi-Cell XR Cell Viability Analyzer (Beckman Coulter, Mississauga, ON, Canada). Paraquat (Paraquat dichloride x-hydrate, Sigma-Aldrich) was dissolved in distilled water and diluted with culture media to prepare specific treatment concentrations. Experiments were performed using serum-free media.

To determine the cytotoxicity of NAC alone, cells were treated with control media or media containing different concentrations of NAC (0 to 50 mM final NAC concentrations). To determine the effect of NAC or L-NAC on the toxicity of PQ, cells were first pretreated with control, empty liposomes- (EL-) NAC-, or L-NAC-containing media (5.0 mM for 4 h), followed by treatment with control or PQ-containing media.

### 2.2. N-Acetylcysteine (NAC) Preparation

NAC (N-acetyl-L-cysteine, SigmaUltra > 99% TLC; Sigma-Aldrich) was dissolved in PBS and adjusted to pH 7.4 to produce a 0.1 M stock solution. Following filter sterilization (0.2 *μ*m pore-size filters), specific volumes of N-acetylcysteine stock solution were added to culture media for the pretreatment/treatment of cells. NAC stocks were made fresh daily.

### 2.3. Liposomal-N-Acetylcysteine Preparation

Liposomal-N-acetylcysteine (L-NAC) was prepared from a mixture of DPPC (dipalmitoylphosphatidylcholine) and NAC in a 7 : 3 molar ratio by using a dehydration-rehydration method as described in [13]. Liposomal vesicle size was determined with a Submicron Particle Sizer (Nicomp Model 270) following rehydration and was found to have a mean diameter of 181.5 ± 19.6 nm. The encapsulation efficiency of NAC by DPPC-liposomes was measured as 18.5%.

### 2.4. Cell Viability

Cell viability was measured with the MTT (3-(4,5-dimethylthiazol-2-yl)-2,5-diphenyltetrazolium bromide) assay as previously described in [26]. Viabilities of challenged cells were assessed relative to control cells.

### 2.5. Determination of Intracellular NAC and PQ Concentration with Ultraperformance Liquid Chromatography (UPLC)

The intracellular levels of NAC, PQ, and GSH were determined by an ultraperformance liquid chromatographic (UPLC) method using a Waters Acquity system equipped with a binary solvent manager, an automated sample manager, and a photodiode array detector (Waters, Milford, Mss, USA) as described previously by Mitsopoulos and Suntres [26]. Briefly, after each treatment, cells were harvested via trypsinization, washed twice with phosphate-buffered saline (PBS), lysed via sonication (20 s, 100% amplitude; Sonic Dismembrator Model 500, Fisher Scientific, Pittsburgh, Pa, USA), centrifuged, and then passed through a 0.2 *μ*m filter. The ultrafiltrate was injected onto an Acquity UPLC HSS T3 analytical column (2.1 mm I.D. × 150 mm length, 1.8 *μ*m particles) with a Vanguard 2.1 mm I.D. × 5 mm length guard column, at 30°C. The mobile phase consisted of 23 mM ammonium formate (pH 3) at a flow rate of 0.250 mL/min. NAC, PQ, and GSH were measured at wavelengths of 200.3, 257.7, and 202.1 nm, respectively, and values were normalized to total protein using the Micro Lowry Total Protein Kit-Peterson's Modification (Sigma-Aldrich), in accordance with the manufacturer's instructions.

### 2.6. Determination of Reactive Oxygen Species Levels

The intracellular levels of reactive oxygen species were determined by staining the cells with CM-H_2_DCFDA (5-(and 6-) chloromethyl-2′,7′-dichlorodihydrofluorescein diacetate, acetyl ester) (Molecular Probes, Eugene, Ore, USA) in PBS as previously described in [26]. Flow cytometric analysis was performed using a BD FACSCalibur Flow Cytometer (BD Biosciences, San Jose, Calif, USA) with BD CellQuest Pro Software. A minimum of 10,000 gated events were acquired per sample.

### 2.7. Measurement of Mitochondrial Membrane Potential

Mitochondrial membrane potential was assessed using the MitoProbe JC-1 Assay Kit for Flow Cytometry (Molecular Probes). Following challenge, cells were washed with PBS and stained for 30 minutes with JC-1 (5,5′,6,6′-tetrachloro-1,1′,3,3′-tetraethylbenzimidazolylcarbocyanine iodide), a cationic dye that exhibits potential-dependent accumulation in mitochondria, under standard incubation conditions. Stained cells were detached from the plate surface and suspended in PBS for flow cytometric analysis using the FL1-H and FL2-H channels of a BD FACSCalibur Flow Cytometer (BD Biosciences) with BD CellQuest Pro software. A minimum of 10,000 gated events were acquired per sample. Mitochondrial depolarization was indicated by decreased red fluorescence intensity due to concentration-dependent formation of red fluorescent J-aggregates.

### 2.8. Gene Expression Analysis

Gene array analysis of cells challenged with 0.25 mM PQ for 4 h following pretreatment with control, NAC-containing or L-NAC-containing media (5.0 mM for 4 h) was performed as detailed previously in [26] using the Human Stress and Toxicity PathwayFinder RT^2^ Profiler PCR Array (Table 1; SA Biosciences).

Conventional RT-PCR analysis of cells challenged as indicated previously was performed using the RT^2^ qPCR Primer Assays (Table 2; SA Biosciences). The methodology was carried out similar to that outlined for the gene arrays with the exception that 1 *μ*L of the appropriate primer was manually added to each well of the iCycler iQ 96-well PCR Plates (Bio-Rad) and was covered with Microseal “B” Film (Bio-Rad).

### 2.9. Measurement of Cytokine Levels

Cells seeded into sterile 25 cm^2^ culture flasks (Corning) at 1.35 × 10^6^ cells/flask were incubated to 80% confluence overnight, then washed with PBS and pretreated with control, NAC-containing or L-NAC-containing media (5.0 mM for 4 h) followed by challenge with control or PQ-containing media (0.25 or 1.0 mM for 4 h). Following incubation, media of treated cells were analyzed for cytokine levels using a Human Grp I Cytokine 7-Plex Panel kit (Bio-Rad) specific for interleukin (IL)-1*β*, IL-6, IL-8, IL-10, IL-15, TNF-*α*, and eotaxin, using a Bio-Plex 200 System (Bio-Rad) with Bio-Plex Manager software in accordance with the manufacturer's instructions.

### 2.10. Statistics

Data are presented as mean ± S.E.M (*n* ≥ 3) and analyzed for statistical significance using the paired Student's *t*-test, with *P* < .05 considered significant. For normalized data, a paired one-sample *t*-test was performed comparing means to a hypothetical mean of 1 (*P* < .05).

## 3. Results

### 3.1. Effect of NAC on Viability of A549 Cells

**Figure 1:**
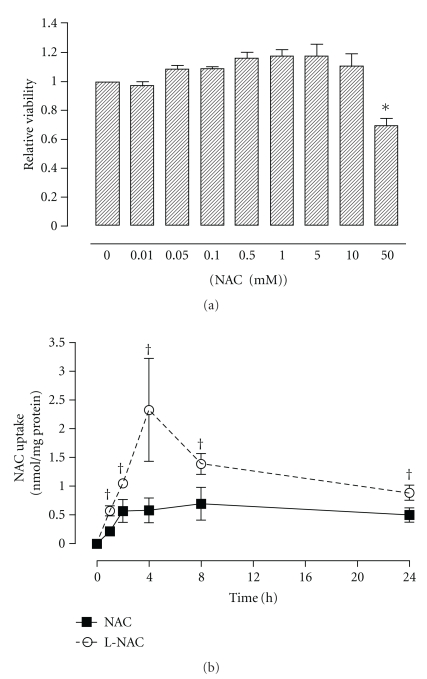
Effect of NAC on the cell viability (a) and uptake of NAC (b) in A549 cells. The viability of cells treated for 24 h with increasing concentrations of NAC was assessed using the MTT assay. Bars represent mean ± S.E.M. of 3 independent experiments performed in octuplet. *denotes significant difference relative to control (*P* < .05). For the uptake studies (b), cells were treated with either 5.0 mM NAC- or L-NAC-containing media for various time-points up to 24 h; intracellular NAC levels were measured with a UPLC method as described in Section 2. (solid line: NAC treatment; dotted line: L-NAC treatment). Data points represent mean ± S.E.M. of 3 independent experiments performed in duplicate. ^†^denotes significant difference relative to NAC-treated group (*P* < .05).

Challenge of A549 cells with NAC at concentrations ranging from 0 to 10 mM did not have any effect on cell viability 24 h after NAC exposure. However, a 30% decrease in viability relative to control cells was observed following treatment with 50.0 mM NAC (Figure 1(a)). A NAC concentration of 5.0 mM was used for all subsequent experiments.

### 3.2. Uptake of NAC in A549 Cells

The uptake of NAC by A549 cells was assessed using UPLC following treatment with 5.0 mM NAC- or L-NAC-containing media for 0, 1, 2, 4, 8, and 24 h (Figure 1(b)). Treatment of A549 cells with conventional NAC resulted in increased NAC uptake up to 2 h posttreatment; thereafter, levels remained unchanged up to 24 h posttreatment. Cells treated with L-NAC exhibited increased uptake over time, with maximal levels achieved following 4 h treatment. Under all investigated conditions, the uptake of NAC by A549 cells was significantly greater following treatment with the L-NAC formulation compared to NAC.

### 3.3. Effect of NAC and L-NAC Pretreatment on Cell Viability after PQ Challenge

**Figure 2:**
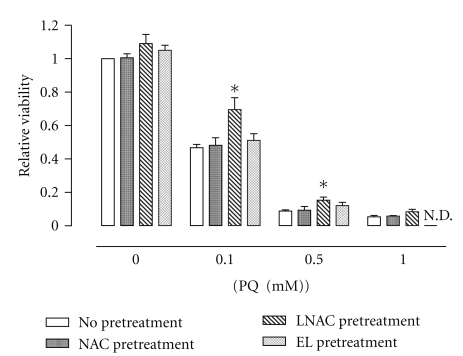
Effect of EL, NAC, or L-NAC pretreatment on viability of PQ-challenged cells. The viability of cells pretreated with control media (no pretreatment), or 5.0 mM NAC- (NAC Pretreatment), L-NAC- (L-NAC pretreatment) or empty liposome-containing media (EL pretreatment) for 4 h prior to 24 h PQ challenge (0, 0.1, 0.5, or 1.0 mM) was assessed using the MTT assay. Bars represent mean ± S.E.M. of 3 independent experiments performed in octuplet. *denotes significant difference relative to cells with no pretreatment (*P* < .05); N.D.: not determined.

Challenge of A549 cells with PQ resulted in concentration-dependent decreases in cell viability (Figure 2). Viability of cells challenged with PQ (0.1 and 0.5 mM) for 24 h was higher in those cells pretreated with L-NAC. In contrast, pretreatment with NAC or empty liposomes (EL) did not confer any observable effect on cell viability of PQ-challenged cells under these conditions.

### 3.4. Effect of NAC and L-NAC Pretreatment on Cellular Redox Status and PQ Uptake following PQ Challenge

**Figure 3:**
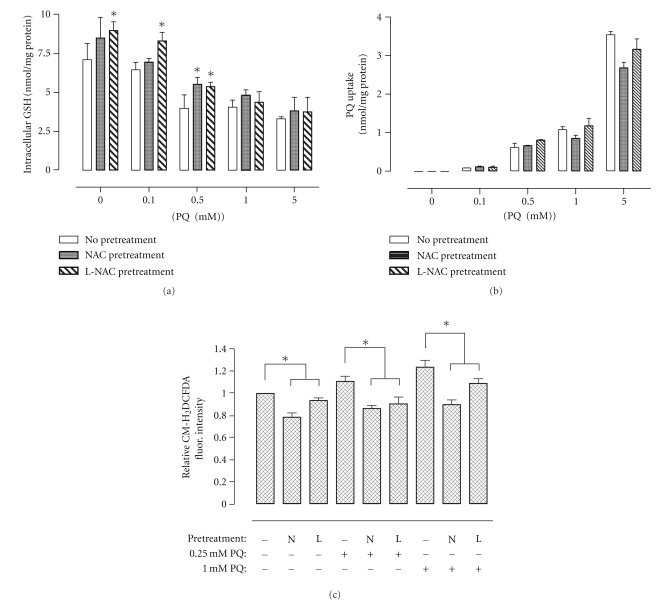
Effect of NAC or L-NAC pretreatment on intracellular levels of GSH (a), PQ (b), and ROS (c) in PQ-challenged cells. Cells pretreated for 4 h with control media (No Pretreatment) or 5.0 mM NAC- (NAC Pretreatment) or L-NAC-containing media (L-NAC Pretreatment) were challenged with increasing PQ concentrations (0, 0.1, 0.5, 1.0, and 5.0 mM) for 24 h. Cells were harvested and lysed for concomitant measurement of intracellular GSH content (a) and PQ uptake (b) via UPLC analysis and normalized to total protein. For the measurement of ROS, cells were stained for 30 min posttreatment with the cell permeable CM-H_2_DCFDA fluorescent dye specific for oxidative species. Adherent cells were scraped and analyzed flow cytometrically using the FL1-H channel. Bars represent mean ± S.E.M. of 3 independent experiments. *denotes significant difference relative to cells with no pretreatment (*P* < .05).

Exposure of cells to increasing concentrations of PQ for 24 h significantly decreased intracellular GSH content, which correlated with increases in cellular PQ uptake, as measured by UPLC (Figure 3). In general, pretreatment with L-NAC but not NAC resulted in lower PQ-induced depletion in the levels of intracellular GSH content of cells. Pretreatment with NAC or L-NAC had no effect on the linear (*R* ^2^ = 0.969) uptake of PQ in A549 cells challenged with increasing concentrations of PQ (Figure 3(b)). ROS levels increased following PQ exposure (Figure 3(c)), but pretreatment with NAC or L-NAC significantly reduced levels of ROS in 0.25 and 1.0 mM PQ-challenged cells (4 h) to either basal or subbasal levels as assessed via flow cytometric analysis of CM-H_2_DCFDA-stained cells (Figure 3(c)).

### 3.5. Effect of NAC or L-NAC Pretreatment on Mitochondrial Membrane Potential following PQ Challenge

**Figure 4:**
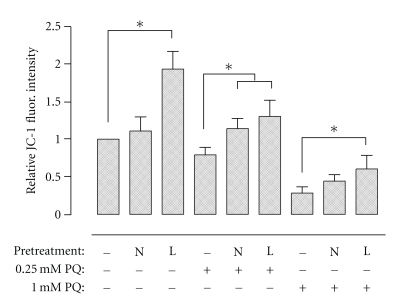
Effect of NAC or L-NAC pretreatment on mitochondrial membrane potential following PQ challenge. Cells pretreated for 4 h with control media or 5.0 mM NAC- (N) or L-NAC-containing media (L) were challenged with 0, 0.25, or 1.0 mM PQ for 4 h. Cells were stained for 30 min posttreatment with the cell permeable JC-1 fluorescent dye. Bars represent mean ± S.E.M. of 3 independent experiments. *denotes significant difference relative to cells with no pretreatment (*P* < .05).

The mitochondrial membrane potential of cells challenged with 0.25 mM PQ for 4 h was significantly decreased relative to untreated control cells and was further decreased following 1.0 mM PQ challenge. Pretreatment with L-NAC was effective in preventing the decreases of mitochondrial membrane potential in both 0.25 and 1.0 mM PQ-challenged cells, returning it to basal levels in the former, as well as increasing it nearly 2-fold when compared to untreated control cells (Figure 4). Conversely, pretreatment with NAC only significantly prevented the decrease in mitochondrial membrane potential of 0.25 mM PQ-challenged cells, having no apparent effect on untreated control or 1.0 mM PQ-challenged cells.

### 3.6. Effect of NAC or L-NAC Pretreatment on the Secretion of Inflammatory Cytokines after PQ Challenge

**Figure 5:**
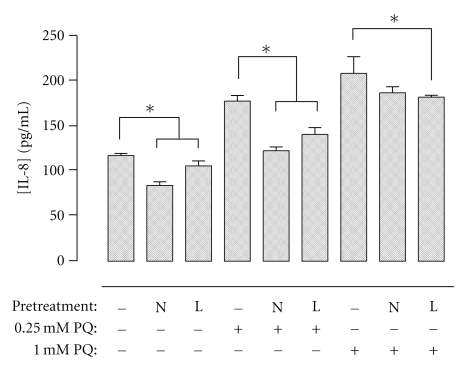
Effect of NAC or L-NAC pretreatment on IL-8 levels after PQ exposure. Cells pretreated for 4 h with control media or 5.0 mM NAC- (N) or L-NAC-containing media (L) were challenged with 0, 0.25, or 1.0 mM PQ for 4 h. Cell culture supernatants were collected immediately following challenge and concomitantly analyzed for IL-8 using the Bio-Plex suspension array system. Bars represent mean ± S.E.M. of 3 independent experiments. *denotes significant difference relative to cells with no pretreatment (*P* < .05).

Levels of IL-8 secreted by cells exposed to 0.25 mM and 1.0 mM PQ were significantly increased relative to untreated control cells (Figure 5). Both NAC and L-NAC pretreatments decreased IL-8 levels in untreated control cells and 0.25 mM PQ-challenged cells, while L-NAC, but not NAC, was also able to significantly reduce levels of IL-8 following 1.0 mM PQ challenge. Levels of IL-1*β*, IL-6, IL-10, IL15, TNF-*α*, and eotaxin were not reliably detectable under these conditions.

### 3.7. Effect of NAC or L-NAC Pretreatment on Cellular Gene Expression following PQ Challenge

**Figure 6:**
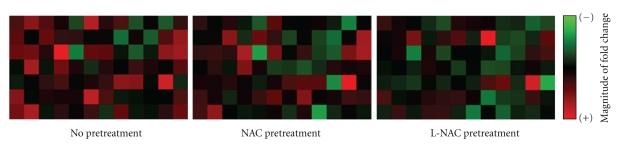
Effect of NAC or L-NAC pretreatment on the magnitude of gene expression in PQ-challenged cells. RNA was extracted from cells challenged with 0 or 0.25 mM PQ for 4 h following pretreatment with 5.0 mM NAC- or L-NAC-containing media and analyzed via quantitative reverse-transcription PCR using a gene array. The magnitude of expression of each gene is expressed on a scale ranging from minimal (intense green) to maximal (intense red) expression (*n* = 3 independent experiments).

Changes in gene expression were assessed using a gene array designed to study genes involved with cellular stress and toxicity. The magnitude of gene expression in cells pretreated with NAC or L-NAC prior to 0.25 mM PQ challenge for 4 h was generally decreased relative to challenged cells with no pretreatment (Figure 6). Fold changes (relative to control cells) of each gene of the array following PQ challenge with no pretreatment, NAC pretreatment, or L-NAC pretreatment are listed in Table 1. 

The expression of many oxidative or metabolic stress-related genes was not significantly altered under any of the studied conditions, with the exception of CYP1A1 and PTGS1 being significantly upregulated in PQ-challenged cells pretreated with L-NAC. The expression of all studied heat shock genes remained more or less unchanged in PQ-treated cells but HSPA2, HSPA4, and HSPA1L were each significantly downregulated with NAC or L-NAC pretreatment. The expression of EGR1 was increased 2.0-fold in PQ challenged cells in the absence of antioxidant pretreatment but its expression was maintained at control levels with L-NAC pretreatment. 

Antioxidant pretreatment of cells subsequently challenged with PQ had an effect on genes related to growth arrest and senescence as well. Briefly, GDF15 and DDIT3 were both significantly upregulated 1.9-fold following PQ challenge and were modulated with L-NAC (1.4- and 1.5-fold, resp.), but not NAC (2.0- and 2.2-fold, resp.), pretreatment. CDKN1A was upregulated 1.5-fold following PQ challenge and was progressively upregulated further with both NAC (1.7-fold) and L-NAC (1.9-fold) pretreatment. Also significantly altered were the expression patterns of several inflammatory genes. IL18 was upregulated 1.5-fold in PQ-challenged cells, but its expression was modulated with NAC or L-NAC pretreatment. Using individual primer assays with conventional RT-PCR, we found IL8 to be significantly upregulated (2.2-fold) in PQ-challenged cells with no pretreatment, but it was progressively modulated with NAC (2.0-fold) and L-NAC (1.7-fold) pretreatment. It is worth noting that the IL10 gene, which codes for the anti-inflammatory cytokine IL-10, was down-regulated in PQ-challenged cells, an effect reversed by L-NAC, but not NAC, pretreatment. Finally, the expression of many apoptosis signalling genes was not altered under the studied conditions with the exception of CASP10, which was upregulated 1.7-fold in PQ-challenged cells with no pretreatment and was modulated by both NAC and L-NAC pretreatment. The effect of L-NAC was confirmed to not be a direct result of the lipids composing the liposomes as pretreatment with empty liposomes did not alter the expression compared to PQ-challenged cells with no pretreatment (Table 2). 

Conventional RT-PCR assays were performed to validate findings obtained from the gene arrays. Similar gene expression patterns were observed for the majority of the genes (e.g., CAT, CYP1A1, IL1A, NFKBIA, and SOD1) analyzed by both methods.

### 3.8. Validation of Gene Array Data Using Conventional RT-PCR

**Figure 7:**
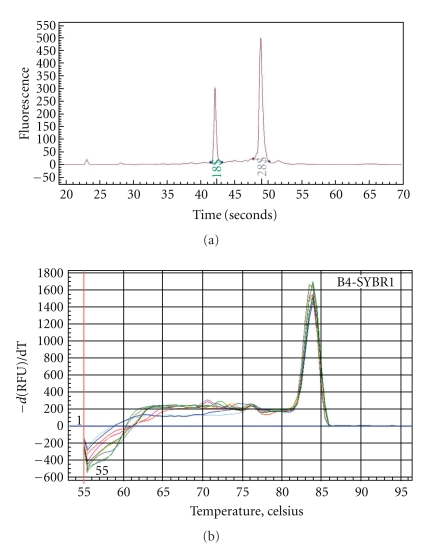
Validation of RNA integrity (a) and assessment of PCR gene product quality (b). Aliquots of extracted RNA from control or PQ-challenged A549 cells with or without pretreatment were assessed for RNA concentration and integrity using the Experion automated electrophoresis station. A representative electropherogram displays 18 and 28 S rRNA peaks. Representative first-derivative dissociation curves of amplified PCR product of 4 h control and PQ-challenged cells pretreated with control media or NAC- or L-NAC-containing media are depicted.

Figure 7 depicts a representative electropherogram of extracted RNA, achieved using the Experion automated electrophoresis station, indicating high RNA integrity with little or no apparent degradation of 18 and 28 S rRNA. Additionally, a single peak (or zero if no product was amplified) was present in first-derivative dissociation curves for every PCR reaction on all gene arrays and conventional RT-PCR assays, indicating that only a single PCR product (i.e., the gene of interest) was amplified in each case.

### 3.9. Effects of Empty Liposomes on Cytotoxicity and Gene Array Analysis

Challenge of A549 cells with empty liposomes was not toxic to cells. Also, pretreatment of cells with empty liposomes did not confer any protection against PQ-induced cytotoxicity (Figure 2) and gene array analysis (Table 2).

## 4. Discussion

The results of the present study showed that exposure of A549 cells to PQ *in vitro* resulted in a concentration- and time-dependent accumulation of PQ which was associated with concomitant increases in the intracellular levels of ROS and decreases in GSH levels, confirming results from other studies that PQ exerts its toxic effects, to a major extent, via oxidative stress mechanisms [1–3, 26]. Accordingly, research efforts in the management of PQ poisoning are also directed towards the use of antioxidants. A potential antioxidant candidate is NAC because not only it is available in the clinic but also its thiol group provides free radical-scavenging properties and it acts as a source of cysteine, often the limiting precursor of *de novo* GSH synthesis [3, 17, 18].

In order to assess the cytoprotective effects of both the conventional and liposomal NAC formulations, we first investigated their optimal treatment conditions in A549 cells. NAC has been reported to exhibit cytotoxicity at variable concentrations depending on cell type: 10 mM NAC was nontoxic in human bronchial epithelial cells [17], 40 mM was nontoxic in 3T3 fibroblasts [27], and 50 mM was nontoxic in aortic endothelial cells [17]; however, 30 mM was cytotoxic in vascular smooth muscle cells, monocytes, and neutrophils, and as low as 5.0 mM NAC was cytotoxic in porcine aortic endothelial cells [17]. Studies involving NAC in A549 cells have generally employed concentrations ranging from 1 to 10 mM [26, 28–31]. Our results indicate that concentrations of 10.0 mM or less had no negative impact on cell viability, while a greater concentration (i.e., 50.0 mM) resulted in significantly decreased cell viability after 24 h (Figure 1). 

Both NAC formulations conferred protection against PQ-induced cytotoxicity but generally L-NAC was more effective than the conventional NAC formulation in limiting the PQ-induced decreases of cellular GSH content (Figure 3(a)) and production of ROS (Figure 3(c)). The protective effect of NAC or L-NAC cannot be attributed to the effect of the antioxidant formulations on PQ uptake since NAC or L-NAC pretreatment had no effect on intracellular PQ levels in A549 cells (Figure 3(b)). It is unclear whether this protective effect is a result of NAC's direct scavenging properties or the *de novo* synthesis of GSH using NAC as a precursor. The possible reason for the protective effects being more prominent in cells pretreated with L-NAC was the greater and more sustainable intracellular NAC levels that can be achieved via liposomal delivery (Figure 1(b)). Cellular uptake experiments showed that the uptake of NAC as a liposomal formulation was much greater (i.e., up to 4-fold) under all conditions studied when delivered as L-NAC compared to NAC (Figure 1(b)). Thus, the higher and more sustained intracellular NAC levels are responsible for maintaining a normal cellular redox status as evidenced by the reduced production of ROS and higher GSH levels. It should be noted that pretreatment of cells with empty liposomes consisting of DPPC lipids did not alter the PQ-induced changes in cytotoxicity and gene expression. 

The mitochondria are thought to be essential targets of PQ and important in its toxicity. In fact, there is evidence that PQ disrupts the mitochondrial electron transfer chain resulting in a reduction of metabolic function, and it is suggested that lesions due to PQ first occur in the mitochondria [32–34]. Pretreatment with NAC or L-NAC exhibited a beneficial effect on the mitochondrial membrane potential of PQ-challenged cells (Figure 4). NAC or L-NAC pretreatment increased the membrane potential above control levels in 0.25 mM PQ-challenged cells for 4 h, while L-NAC, but not NAC, pretreatment limited the decrease of membrane potential in cells exposed to 1.0 mM PQ. Interestingly, control cells pretreated with L-NAC exhibited nearly a 2-fold increase in fluorescence intensity relative to control cells with no pretreatment. Suntres et al. [8] have shown that radioactively-labelled liposomal antioxidant vesicles were associated with mitochondria, and antioxidants that are selectively accumulated into mitochondria can inhibit mitochondrial oxidative damage that contributes to a range of degenerative diseases related to oxidative stress. 

 The maintenance of cellular redox status is crucial for cellular homeostasis, and its dysregulation towards a more oxidized intracellular environment is associated with aberrant transcriptional activation and gene expression affecting several processes such as cell growth, differentiation, and inflammation [22, 35–37]. In this study, the magnitude of PQ-induced changes in gene expression in cells pretreated with NAC or L-NAC prior to 0.25 mM PQ challenge for 4 h was generally lower with the L-NAC being a more effective treatment than NAC (Figure 6). Although the exact mechanism(s) by which NAC affected the pathways involved in signal transduction and gene expression cannot be delineated from the results of this study, it is possible that these pathways are regulated by oxidants and redox-sensitive steps since increasing levels of intracellular NAC affect the steady state level of oxidants (Figure 3(c)) and can modify the redox status of the cell (Figure 3), an effect known to exert a regulatory effect on transcriptional activation and gene expression [22, 35, 38–40]. For example, expression of GDF15, IL8, EGR1, and CASP10 genes is known to be upregulated in oxidative stress-induced conditions, a treatment effect counteracted by the presence of antioxidants [36, 41–47]. The upregulation of GDF15, a protein that plays a role in regulating inflammatory and apoptotic pathways in injured tissues and during disease processes [48–50], and EGR1, which encodes a transcriptional regulator that activates genes (including p53) required for differentiation and mitogenesis [51], was maintained closer to normal levels in cells pretreated with L-NAC, but not NAC (Table 1). The expression of CASP10, which codes for the initiator caspase-10 involved in the death-inducing signaling complex during apoptosis (Bidere et al. [52]), was upregulated 1.7-fold in PQ-challenged cells but was closer to control levels in PQ-challenged cells pretreated with NAC or L-NAC (Table 1). It is evident that the preservation of cellular homeostasis by L-NAC, and to a lesser extent by NAC, promoted cell survival. 

Paraquat administration has been shown to be associated with an infiltration of neutrophils in lung [5, 53]. Interleukin-8 (IL-8), a potent proinflammatory cytokine released in response to injury, has a key role in the recruitment and activation of neutrophils during inflammation [54, 55]. The expression of IL8 gene was upregulated in PQ-challenged cells, but its expression was substantially modulated with pretreatment of L-NAC and to a lesser extent with NAC (Table 2). The inhibitory effects of NAC and L-NAC on IL-8 expression were correlated with comparable changes in IL-8 protein secretion in the cell culture supernatant of PQ-challenged cells (Figure 5). The ability of NAC to modulate the upregulation of IL-8 has been described in other studies as well: the increased IL-8 gene [56] and protein [57, 58] expression of PQ-challenged peripheral blood mononuclear cells was blocked by NAC [57], and NAC administration was found to inhibit the release of chemotactic factors for neutrophils and consequently reduce their infiltration into the lungs of PQ-challenged rats [9].

Oxidative stress, which occurs when the redox homeostasis within the cell is altered, is a key pleiotropic modulator which may be involved in the upregulation and/or downregulation of several genes [35, 37]. IL10, which codes for the anti-inflammatory cytokine interleukin-10 (IL-10), is down-regulated 2.2-fold following PQ-treatment (Table 2), suggesting that the cell may be actively repressing anti-inflammatory mediators in favor of proinflammatory mediators (e.g., IL-8). The ability of NAC to prevent the downregulation of IL-10, being greater in cells pretreated with L-NAC than NAC, is an evidence to confirm that the higher intracellular levels of NAC maintain the redox status of cells closer to normalcy. 

In conclusion, the results of the present study suggest that pretreatment of A549 cells with NAC, both in its conventional and liposomal form, conferred cytoprotection against PQ-induced toxicity. This was mainly attributed to its ability to ameliorate cellular redox status (i.e., intracellular GSH content and ROS levels) and was independent of PQ uptake. These protective effects were more evident in cells pretreated with L-NAC, which is attributed, at least in part, to the increased NAC levels achieved via liposomal delivery.

##  Conflict of Interests

The authors declare that there is no conflict of interest.

## Figures and Tables

**Table 1:** Relative expression, via microarray analysis, of genes involved with cellular stress and toxicity in cells challenged with 0.25 mM PQ for 4 h following pretreatment with control, NAC-containing, or L-NAC-containing media. Genes are listed in order of decreasing fold change in cells pretreated with control media and challenged with PQ. Fold change is expressed relative to untreated control cells using the housekeeping genes B2M, HPRT1, RPL13A, and GAPDH. *Note*. the proposed housekeeping gene ACTB was significantly altered following PQ challenge, and was not used in this capacity in our study. *n* = 3 independent experiments.

GeneBank accession no.	Gene name	Symbol	Fold change					
			Control media + PQ	*P* value	NAC + PQ	*P* value	LNAC + PQ	*P* value
*Oxidative/metabolic stress*								
NM_005953	Metallothionein 2A	MT2A	1.17	0.127	−1.07	0.616	1.33	0.229
NM_002133	Heme oxygenase (decycling) 1	HMOX1	1.14	0.531	1.34	0.170	−1.16	0.456
NM_000962	Prostaglandin-endoperoxide synthase 1 (prostaglandin G/H synthase and cyclooxygenase)	PTGS1	1.10	0.671	1.34	0.208	1.50	0.026**
NM_001885	Crystallin, alpha B	CRYAB	1.07	0.847	1.36	0.190	1.18	0.510
NM_002574	Peroxiredoxin 1	PRDX1	1.07	0.505	1.05	0.367	1.04	0.662
NM_000454	Superoxide dismutase 1, soluble (amyotrophic lateral sclerosis 1 (adult))	SOD1	1.04	0.343	1.05	0.332	−1.07	0.200
NM_000581	Glutathione peroxidase 1	GPX1	1.01	0.812	1.05	0.395	−1.08	0.286
NM_000849	Glutathione S-transferase M3 (brain)	GSTM3	−1.02	0.833	−1.09	0.600	−1.03	0.743
NM_001752	Catalase	CAT	−1.03	0.740	−1.31	0.136	−1.16	0.168
NM_000637	Glutathione reductase	GSR	−1.08	0.594	−1.57	0.098	−1.46	0.111
NM_005809	Peroxiredoxin 2	PRDX2	−1.12	0.528	−1.01	0.892	−1.22	0.573
NM_001461	Flavin-containing monooxygenase 5	FMO5	−1.28	0.154	−1.53	0.072	−1.60	0.065
NM_000499	Cytochrome P450, family 1, subfamily A, polypeptide 1	CYP1A1	−1.40	0.057	1.09	0.583	4.44	0.003**
NM_000941	P450 (cytochrome) oxidoreductase	POR	−1.93	0.174	1.26	0.367	−2.70	0.122
NM_001979	Epoxide hydrolase 2, cytoplasmic	EPHX2	−2.06	0.066	−2.24	0.225	−1.22	0.369
NM_000773	Cytochrome P450, family 2, subfamily E, polypeptide 1	CYP2E1	—	—	—	—	—	—
NM_000780	Cytochrome P450, family 7, subfamily A, polypeptide 1	CYP7A1	—	—	—	—	—	—
NM_002021	Flavin-containing monooxygenase	FMO1	—	—	—	—	—	—

*Heat shock*								
NM_005347	Heat shock 70 kDa protein 5 (glucose-regulated protein, 78 kDa)	HSPA5	1.35	0.004**	1.24	0.026**	−1.00	0.983
NM_007034	DnaJ (Hsp40) homolog, subfamily B, member 4	DNAJB4	1.32	0.052	1.10	0.568	−1.02	0.813
NM_001539	DnaJ (Hsp40) homolog, subfamily A, member 1	DNAJA1	1.29	0.048**	1.12	0.439	−1.08	0.443
NM_005526	Heat shock transcription factor 1	HSF1	1.14	0.074	1.08	0.253	1.13	0.354
NM_006644	Heat shock 105 kDa/110 kDa protein 1	HSPH1	1.14	0.253	1.03	0.551	1.03	0.612
NM_001 040 141	Heat shock protein 90 kDa alpha (cytosolic), class A member 2	HSP90AA2	1.08	0.200	1.05	0.557	1.09	0.279
NM_002157	Heat shock 10 kDa protein 1 (chaperonin 10)	HSPE1	1.07	0.414	−1.06	0.367	−1.07	0.503
NM_002156	Heat shock 60 kDa protein 1 (chaperonin)	HSPD1	1.06	0.671	−1.15	0.312	−1.08	0.406
NM_006597	Heat shock 70 kDa protein 8	HSPA8	1.02	0.922	−1.09	0.599	−1.33	0.060
NM_021979	Heat shock 70 kDa protein 2	HSPA2	1.01	0.908	−1.40	0.004**	−1.46	0.003**
NM_002154	Heat shock 70 kDa protein 4	HSPA4	−1.06	0.523	−1.47	0.036**	−1.71	0.004**
NM_007355	Heat shock protein 90 kDa alpha (cytosolic), class B member 1	HSP90AB1	−1.07	0.666	1.00	0.995	−1.61	0.058
NM_005345	Heat shock 70 kDa protein 1A	HSPA1A	−1.09	0.478	−1.29	0.102	1.00	0.957
NM_001540	Heat shock 27 kDa protein 1	HSPB1	−1.24	0.227	−1.00	0.924	−1.28	0.218
NM_005527	Heat shock 70 kDa protein 1-like	HSPA1L	−1.29	0.084	−1.65	0.032**	−1.42	0.077
NM_002155	Heat shock 70 kDa protein 6 (HSP70B′)	HSPA6	—	—	—	—	—	—

*Proliferation/carcinogenesis*								
NM_001964	Early growth response 1	EGR1	1.97	0.039**	1.70	0.117	−1.24	0.395
NM_005190	Cyclin C	CCNC	1.30	0.226	1.02	0.995	1.18	0.561
NM_182649	Proliferating cell nuclear antigen	PCNA	1.14	0.283	−1.08	0.461	−1.04	0.678
NM_053056	Cyclin D1	CCND1	−1.03	0.885	−1.28	0.225	−1.02	0.803
NM_004060	Cyclin G1	CCNG1	−1.05	0.410	−1.02	0.316	1.07	0.435
NM_005225	E2F transcription factor 1	E2F1	−1.28	0.473	−1.16	0.450	−1.94	0.107

*Growth arrest/senescence*								
NM_004864	Growth differentiation factor 15	GDF15	1.91	0.000**	1.96	0.003**	1.37	0.045**
NM_004083	DNA-damage-inducible transcript 3	DDIT3	1.87	0.000**	2.17	0.000**	1.46	0.020**
NM_000389	Cyclin-dependent kinase inhibitor 1A (p21, Cip1)	CDKN1A	1.50	0.002**	1.72	0.001**	1.88	0.000**
NM_001924	Growth arrest and DNA-damage-inducible, alpha	GADD45A	1.29	0.125	1.50	0.044**	−1.01	0.942
NM_002392	Mdm2, transformed 3T3 cell double minute 2, p53-binding protein (mouse)	MDM2	1.26	0.119	−1.01	0.999	1.25	0.112
NM_000546	Tumor protein p53	TP53	1.14	0.294	−1.10	0.546	−1.11	0.353
NM_002178	Insulin-like growth factor-binding protein 6	IGFBP6	−1.19	0.186	1.04	0.761	−1.58	0.032**

*Inflammatory*								
NM_001562	Interleukin 18 (interferon-gamma-inducing factor)	IL18	1.49	0.010**	1.26	0.106	1.28	0.016**
NM_000575	Interleukin 1, alpha	IL1A	1.47	0.085	1.18	0.481	1.88	0.020**
NM_000602	Serpin peptidase inhibitor, clade E (nexin, plasminogen activator inhibitor type 1), member 1	SERPINE1	1.38	0.035**	1.34	0.096	1.01	0.976
NM_000595	Lymphotoxin alpha (TNF superfamily, member 1)	LTA	1.22	0.458	1.38	0.141	−2.19	0.060
NM_003998	Nuclear factor of kappa light polypeptide gene enhancer in B cells 1 (p105)	NFKB1	1.22	0.004**	1.12	0.092	1.36	0.000**
NM_002415	Macrophage migration inhibitory factor (glycosylation-inhibiting factor)	MIF	1.02	0.505	1.13	0.043**	−1.06	0.331
NM_000576	Interleukin 1, beta	IL1B	−1.02	0.948	−1.69	0.357	−1.02	0.936
NM_002989	Chemokine (C-C motif) ligand 21	CCL21	—	—	—	—	—	—
NM_002983	Chemokine (C-C motif) ligand 3	CCL3	—	—	—	—	—	—
NM_002984	Chemokine (C-C motif) ligand 4	CCL4	—	—	—	—	—	—
NM_001565	Chemokine (C-X-C motif) ligand 10	CXCL10	—	—	—	—	—	—

*DNA damage/repair*								
NM_000051	Ataxia telangiectasia mutated	ATM	1.21	0.318	−1.38	0.197	−1.07	0.727
NM_005431	X-ray repair complementing defective repair in Chinese hamster cells 2	XRCC2	1.19	0.125	−1.26	0.261	1.10	0.303
NM_003362	Uracil-DNA glycosylase	UNG	1.11	0.315	−1.01	0.882	−1.01	0.890
NM_000122	Excision repair cross-complementing rodent repair deficiency, complementation group 3 (xeroderma pigmentosum group B complementing)	ERCC3	1.07	0.612	−1.26	0.271	−1.20	0.229
NM_005053	RAD23 homolog A (*S. cerevisiae*)	RAD23A	1.04	0.764	−1.00	0.956	−1.23	0.265
NM_007194	CHK2 checkpoint homolog (*S. pombe*)	CHEK2	1.03	0.844	−1.32	0.111	−1.23	0.100
NM_001923	Damage-specific DNA-binding protein 1, 127 kDa	DDB1	−1.11	0.403	−1.26	0.241	−1.54	0.041**
NM_001983	Excision repair cross-complementing rodent repair deficiency, complementation group 1 (includes overlapping antisense sequence)	ERCC1	−1.16	0.463	1.08	0.666	−1.48	0.278
NM_006297	X-ray repair complementing defective repair in Chinese hamster cells 1	XRCC1	−1.25	0.235	−1.29	0.226	−1.64	0.071
NM_007120	UDP glucuronosyltransferase 1 family, polypeptide A4	UGT1A4	−1.30	0.319	−2.04	0.071	−1.50	0.283

*Apoptosis signalling*								
NM_001230	Caspase 10, apoptosis-related cysteine peptidase	CASP10	1.71	0.006**	1.25	0.194	1.35	0.110
NM_001154	Annexin A5	ANXA5	1.42	0.025**	1.17	0.073	1.42	0.004**
NM_020529	Nuclear factor of kappa light polypeptide gene enhancer in B-cells inhibitor, alpha	NFKBIA	1.20	0.097	1.35	0.014**	1.47	0.017**
NM_001228	Caspase 8, apoptosis-related cysteine peptidase	CASP8	1.15	0.184	−1.08	0.571	1.36	0.017**
NM_004324	BCL2-associated X protein	BAX	−1.04	0.656	−1.12	0.503	−1.30	0.069
NM_003810	Tumor necrosis factor (ligand) superfamily, member 10	TNFSF10	−1.09	0.788	−1.15	0.444	−1.53	0.110
NM_033292	Caspase 1, apoptosis-related cysteine peptidase (interleukin 1, beta, convertase)	CASP1	−1.11	0.818	−1.24	0.150	−1.19	0.627
NM_001065	Tumor necrosis factor receptor superfamily, member 1A	TNFRSF1A	−1.29	0.235	−1.15	0.610	−1.64	0.081
NM_138578	BCL2-like 1	BCL2L1	−1.46	0.028**	−1.37	0.257	−1.42	0.340

*Housekeeping*								
NM_001101	Actin, beta	ACTB	1.35	0.018**	−1.17	0.251	1.23	0.152
NM_004048	Beta-2-microglobulin	B2M	—	—	—	—	—	—
NM_000194	Hypoxanthine phosphoribosyltransferase 1 (Lesch-Nyhan syndrome)	HPRT1	—	—	—	—	—	—
NM_012423	Ribosomal protein L13a	RPL13A	—	—	—	—	—	—
NM_002046	Glyceraldehyde-3-phosphate dehydrogenase	GAPDH	—	—	—	—	—	—

**P < .05.

**Table 2:** Relative expression, via conventional RT-PCR analysis, of genes involved with cellular stress and toxicity in cells challenged with 0.25 mM PQ for 4 h following pretreatment with control on NAC-, L-NAC- or EL-containing media. Fold change is expressed relative to the respective untreated time control using the housekeeping gene RPL13A. *n* = 3 independent experiments performed in triplicate.

GeneBank accession no.	Gene name	Symbol	Fold change							
			Control media + PQ	*P*-value	NAC + PQ	*P*-value	LNAC + PQ	*P*-value	EL + PQ	*P*-value
*Miscellaneous genes*										
NM_00584	Interleukin 8	IL8	2.23	0.007**	2.01	0.001**	1.74	0.010**	2.15	0.009**
NM_002746	Mitogen-activated protein kinase 3	MAPK3	1.47	0.404	1.45	0.472	1.86	0.151	1.55	0.378
NM_002228	Jun oncogene	JUN	1.40	0.240	1.63	0.049**	1.36	0.174	1.09	0.606
NM_002750	Mitogen-activated protein kinase 8	MAPK8	1.18	0.847	1.45	0.544	1.03	0.907	−1.08	0.820
NM_000660	Transforming growth factor, beta 1	TGFB1	1.13	0.593	1.34	0.245	1.26	0.316	1.07	0.664
NM_005252	V-fos FBJ murine osteosarcoma viral oncogene homolog	FOS	1.02	0.866	1.63	0.417	1.09	0.958	1.04	0.737
NM_001315	Mitogen-activated protein kinase 14	MAPK14	−1.03	0.781	1.24	0.590	−1.01	0.930	−1.33	0.911
NM_000572	Interleukin 10	IL10	−2.15	0.079	−1.82	0.126	1.09	0.889	−1.60	0.177

*Verification Genes*										
NM_000576	Interleukin 1, beta	IL1B	1.35	0.677	1.73	0.476	1.77	0.452	1.40	0.656
NM_020529	Nuclear factor of kappa light polypeptide gene enhancer in B-cells inhibitor, alpha	NFKBIA	1.31	0.271	1.46	0.179	1.69	0.087	1.55	0.205
NM_000499	Cytochrome P450, family 1, subfamily A, polypeptide 1	CYP1A1	1.12	0.950	1.10	0.925	2.26	0.155	1.77	0.400
NM_000575	Interleukin 1, alpha	IL1A	1.08	0.802	1.48	0.308	1.65	0.317	1.36	0.446
NM_000594	Tumor necrosis factor (TNF superfamily, member 2)	TNF	−1.00	0.942	1.34	0.255	1.32	0.134	1.36	0.098
NM_000454	Superoxide dismutase 1, soluble (amyotrophic lateral sclerosis 1 (adult))	SOD1	−1.04	0.962	1.02	0.784	−1.43	0.263	−1.24	0.297
NM_001752	Catalase	CAT	−1.12	0.852	−1.21	0.402	−1.30	0.299	−1.25	0.466

**P < .05.
